# Fixed Points of Contractive Mappings in *b*-Metric-Like Spaces

**DOI:** 10.1155/2014/471827

**Published:** 2014-03-30

**Authors:** Nawab Hussain, Jamal Rezaei Roshan, Vahid Parvaneh, Zoran Kadelburg

**Affiliations:** ^1^Department of Mathematics, King Abdulaziz University, P.O. Box 80203, Jeddah 21589, Saudi Arabia; ^2^Department of Mathematics, Qaemshahr Branch, Islamic Azad University, Qaemshahr, Iran; ^3^Young Researchers and Elite Club, Kermanshah Branch, Islamic Azad University, Kermanshah, Iran; ^4^Faculty of Mathematics, University of Belgrade, Studentskitrg 16, 11000 Beograd, Serbia

## Abstract

We discuss topological structure of *b*-metric-like spaces and demonstrate a fundamental lemma for the convergence of sequences. As an application we prove certain fixed point results in the setup of such spaces for different types of contractive mappings. Finally, some periodic point results in *b*-metric-like spaces are obtained. Two examples are presented in order to verify the effectiveness and applicability of our main results.

## 1. Introduction

There are a lot of generalizations of the concept of metric space. The concepts of *b*-metric space and partial metric space were introduced by Czerwik [1] and Matthews [2], respectively. Combining these two notions, Shukla [3] introduced another generalization which is called a partial *b*-metric space.

On the other hand, Amini-Harandi [4] introduced a new extension of the concept of partial metric space, called a metric-like space. The concept of *b*-metric-like space which generalizes the notions of partial metric space, metric-like space, and *b*-metric space was introduced by Alghamdi et al. in [5]. They established the existence and uniqueness of fixed points in a *b*-metric-like space as well as in a partially ordered *b*-metric-like space. In addition, as an application, they derived some new fixed point and coupled fixed point results in partial metric spaces, metric-like spaces, and *b*-metric spaces (see also [6–14]).

The aim of this paper is to examine more closely the topological structure of these spaces. In this context, we demonstrate a fundamental lemma for the convergence of sequences in *b*-metric-like spaces and by using it we prove some fixed point results in the setup of such spaces. Finally, some periodic point results in *b*-metric-like spaces are obtained. Two examples are presented in order to verify the effectiveness and applicability of our main results.

## 2. Preliminaries

### 2.1. Definitions and Basic Properties of Certain Types of Spaces

To clarify the issue, we first recall definitions of *b*-metric, partial metric, partial *b*-metric, and metric-like spaces.

**Definition 1 (see [1])**

Let *X* be a nonempty set and *s* ≥ 1 a given real number. A function *d* : *X* × *X* → *R* ^+^ is a *b*-metric if, for all *x*, *y*, *z* ∈ *X*, the following conditions are satisfied:

(b_1_) *d*(*x*, *y*) = 0 if and only if *x* = *y*,
(b_2_) *d*(*x*, *y*) = *d*(*y*, *x*),
(b_3_) *d*(*x*, *y*) ≤ *s*[*d*(*x*, *z*) + *d*(*z*, *y*)].

The pair (*X*, *d*) is called a *b*-metric space with coefficient *s*.

**Definition 2 (see [2])**

A partial metric on a nonempty set *X* is a mapping *p* : *X* × *X* → ℝ^+^ such that for all *x*, *y*, *z* ∈ *X*

(p_1_) *x* = *y* if and only if *p*(*x*, *x*) = *p*(*x*, *y*) = *p*(*y*, *y*),
(p_2_) *p*(*x*, *x*) ≤ *p*(*x*, *y*),
(p_3_) *p*(*x*, *y*) = *p*(*y*, *x*),
(p_4_) *p*(*x*, *y*) ≤ *p*(*x*, *z*) + *p*(*z*, *y*) − *p*(*z*, *z*).

A partial metric space is a pair (*X*, *p*) such that *X* is a nonempty set and *p* is a partial metric on *X*.

**Definition 3 (see [3])**

A partial *b*-metric on a nonempty set *X* is a mapping *p*_*b*_ : *X* × *X* → ℝ^+^ such that for some real number *s* ≥ 1 and all *x*, *y*, *z* ∈ *X*

(p__*b*_1_) *x* = *y* if and only if *p*_*b*_(*x*, *x*) = *p*_*b*_(*x*, *y*) = *p*_*b*_(*y*, *y*),
(p__*b*_2_) *p*_*b*_(*x*, *x*) ≤ *p*_*b*_(*x*, *y*),
(p__*b*_3_) *p*_*b*_(*x*, *y*) = *p*_*b*_(*y*, *x*),
(p__*b*_4_) *p*_*b*_(*x*, *y*) ≤ *s*[*p*_*b*_(*x*, *z*) + *p*_*b*_(*z*, *y*)] − *p*_*b*_(*z*, *z*).

A partial *b*-metric space is a pair (*X*, *p*_*b*_) such that *X* is a nonempty set and *p*_*b*_ is a partial *b*-metric on *X*. The number *s* is called the coefficient of (*X*, *p*_*b*_).

**Definition 4 (see [4])**

A metric-like on a nonempty set *X* is a mapping *σ* : *X* × *X* → ℝ^+^ such that for all *x*, *y*, *z* ∈ *X*

(*σ*1) *σ*(*x*, *y*) = 0 implies *x* = *y*,
(*σ*2) *σ*(*x*, *y*) = *σ*(*y*, *x*),
(*σ*3) *σ*(*x*, *y*) ≤ *σ*(*x*, *z*) + *σ*(*z*, *y*).

The pair (*X*, *σ*) is called a metric-like space.

Every partial metric space is a metric-like space. Below we give some other examples of metric-like spaces.

**Example 5 (see [15])**

Let *X* = [0,1]. Then the mapping *σ*_1_ : *X* × *X* → ℝ^+^ defined by *σ*_1_(*x*, *y*) = *x* + *y* − *xy* is a metric-like on *X*.

**Example 6 (see [15])**

Let *X* = ℝ; then the mappings *σ*_*i*_ : *X* × *X* → ℝ^+^ (*i* ∈ {2,3, 4}), defined by

(1) σ2(x,y)=|x|+|y|+a,σ3(x,y)=|x−b|+|y−b|,σ4(x,y)=x2+y2,

are metric-like on *X*, where *a* ≥ 0 and *b* ∈ ℝ.

**Definition 7 (see [5])**

Let *X* be a nonempty set and *s* ≥ 1 a given real number. A function *σ*_*b*_ : *X* × *X* → ℝ^+^ is *b*-metric-like if, for all *x*, *y*, *z* ∈ *X*, the following conditions are satisfied:

(*σ*_*b*_1) *σ*_*b*_(*x*, *y*) = 0 implies *x* = *y*,
(*σ*_*b*_2) *σ*_*b*_(*x*, *y*) = *σ*_*b*_(*y*, *x*),
(*σ*_*b*_3) *σ*_*b*_(*x*, *y*) ≤ *s*[*σ*_*b*_(*x*, *z*) + *σ*_*b*_(*z*, *y*)].

A *b*-metric-like space is a pair (*X*, *σ*_*b*_) such that *X* is a nonempty set and *σ*_*b*_ is *b*-metric-like on *X*. The number *s* is called the coefficient of (*X*, *σ*_*b*_).

In a *b*-metric-like space (*X*, *σ*_*b*_) if *x*, *y* ∈ *X* and *σ*_*b*_(*x*, *y*) = 0, then *x* = *y*, but the converse may not be true and *σ*_*b*_(*x*, *x*) may be positive for *x* ∈ *X*. It is clear that every partial *b*-metric space is a *b*-metric-like space with the same coefficient *s* and every *b*-metric space is also a *b*-metric-like space with the same coefficient *s*. However, the converses of these facts need not hold.

**Example 8**

Let *X* = ℝ^+^, *p* > 1 a constant, and *σ*_*b*_ : *X* × *X* → ℝ^+^ be defined by

(2) $$\begin{array}{c} \sigma_{b}\left(x,y\right)={\left(x+y\right)}^{p},\;\forall x,y\in X. \end{array}$$

Then (*X*, *σ*_*b*_) is a *b*-metric-like space with coefficient *s* = 2^*p*−1^, but it is not a partial *b*-metric space. Indeed, for any 0 < *y* < *x* we have *σ*_*b*_(*x*, *x*) = (*x*+*x*)^*p*^ > (*x*+*y*)^*p*^ = *σ*_*b*_(*x*, *y*), so (p__*b*_2_) of Definition 3 is not satisfied.

The following propositions help us to construct some more examples of *b*-metric-like spaces.

**Proposition 9**

Let (*X*, *σ*) be a metric-like space and *σ*_*b*_(*x*, *y*) = [*σ*(*x*,*y*)]^*p*^, where *p* > 1 is a real number. Then *σ*_*b*_ is *b*-metric-like with coefficient *s* = 2^*p*−1^.

**Proof**

The proof follows from the fact that (*a*+*b*)^*p*^ ≤ 2^*p*−1^(*a* ^*p*^ + *b* ^*p*^), where *a*, *b* ∈ ℝ^+^.

From the above proposition and Examples 5 and 6 we have the following examples of *b*-metric-like spaces.

**Example 10**

Let *X* = [0,1]. Then the mapping *σ*_*b*1_ : *X* × *X* → ℝ^+^ defined by *σ*_*b*1_(*x*, *y*) = (*x*+*y*−*xy*)^*p*^, where *p* > 1 is a real number, is *b*-metric-like on *X* with coefficient *s* = 2^*p*−1^.

**Example 11**

Let *X* = ℝ. Then the mappings *σ*_*bi*_ : *X* × *X* → ℝ^+^ (*i* ∈ {2,3, 4}), defined by

(3) σb2(x,y)=(|x|+|y|+a)p,σb3(x,y)=(|x−b|+|y−b|)p,σb4(x,y)=(x2+y2)p,

are *b*-metric-like on *X*, where *p* > 1, *a* ≥ 0, and *b* ∈ ℝ.

**Proposition 12**

Let *X* be a nonempty set such that *d* and *p*_*b*_ are *b*-metric and partial *b*-metric, respectively, *s* > 1, and *σ* is a metric-like on *X*. Then the mappings *σ*_*bi*_ : *X* × *X* → ℝ^+^ (*i* ∈ {5,6, 7}), defined by

(4) σb5(x,y)=pb(x,y)+d(x,y),σb6(x,y)=σ(x,y)+pb(x,y),σb7(x,y)=σ(x,y)+d(x,y),

for all *x*, *y* ∈ *X* are *b*-metric-like on *X*.

**Proof**

Let (*X*, *p*_*b*_) be a partial *b*-metric space and (*X*, *d*) a *b*-metric space with *s* > 1. Then conditions (*σ*_*b*_1), (*σ*_*b*_2), and (*σ*_*b*_3) are obvious for the function *σ*_*b*5_. For instance, if *x*, *y*, *z* ∈ *X* are arbitrary then, as *p*_*b*_ is partial *b*-metric and *d* is *b*-metric on *X*, we have

(5) σb5(x,y)=pb(x,y)+d(x,y)≤s[pb(x,z)+pb(z,y)]−pb(z,z) +s[d(x,z)+d(z,y)]≤s[pb(x,z)+d(x,z)+pb(z,y)+d(z,y)]=s[σb5(x,z)+σb5(z,y)].

Therefore, (*σ*_*b*_3) is satisfied and so (*X*, *σ*_*b*5_) is a *b*-metric-like space. Similarly, one can show that (*X*, *σ*_*b*6_) and (*X*, *σ*_*b*7_) are *b*-metric-like spaces.

From the above proposition and Examples 5 and 6 we have the following examples.

**Example 13**

Let *X* = [0,1]. Then the mapping *σ*_*b*7_ : *X* × *X* → ℝ^+^ defined by *σ*_*b*7_(*x*, *y*) = *x* + *y* −  *xy* + |*x*−*y*|^*p*^, where *p* > 1 is a real number, is *b*-metric-like on *X* with coefficient *s* = 2^*p*−1^.

**Example 14**

Let *X* = ℝ. Then the mappings *σ*_*bi*_ : *X* × *X* → ℝ^+^ (*i* ∈ {8,9, 10}), defined by

(6) σb8(x,y)=|x|+|y|+a+|x−y|p,σb9(x,y)=|x−b|+|y−b|+|x−y|p,σb10(x,y)=x2+y2+|x−y|p,

are *b*-metric-like on *X* with coefficient *s* = 2^*p*−1^, where *p* > 1, *a* ≥ 0, and *b* ∈ ℝ.

Each *b*-metric-like *σ*_*b*_ on *X* generates a topology *τ*_*σ*_*b*__ on *X* whose base is the family of all open *σ*_*b*_-balls {*B*_*σ*_*b*__(*x*, *ɛ*) : *x* ∈ *X*, *ɛ* > 0}, where *B*_*σ*_*b*__(*x*, *ɛ*) = {*y* ∈ *X* : |*σ*_*b*_(*x*, *y*) − *σ*_*b*_(*x*, *x*)| < *ɛ*} for all *x* ∈ *X* and *ɛ* > 0.

Now, we define the concepts of Cauchy sequence and convergent sequence in a *b*-metric-like space.

**Definition 15 (see [5])**

Let (*X*, *σ*_*b*_) be a *b*-metric-like space with coefficient *s*, and let {*x*_*n*_} be any sequence in *X* and *x* ∈ *X*. Then

(i) the sequence {*x*_*n*_} is said to be convergent to *x* with respect to *τ*_*σ*_*b*__, if lim⁡_*n*→*∞*_ *σ*_*b*_(*x*_*n*_, *x*) = *σ*_*b*_(*x*, *x*);
(ii) the sequence {*x*_*n*_} is said to be a Cauchy sequence in (*X*, *σ*_*b*_) if lim⁡_*n*,*m*→*∞*_ *σ*_*b*_(*x*_*n*_, *x*_*m*_) exists and is finite;
(iii) (*X*, *σ*_*b*_) is said to be a complete *b*-metric-like space if for every Cauchy sequence {*x*_*n*_} in *X* there exists *x* ∈ *X* such that
  (7) $$\begin{array}{c} \underset{n,m\to \infty }{\lim}\sigma_{b}\left(x_{n},x_{m}\right)=\underset{n\to \infty }{\lim}\sigma_{b}\left(x_{n},x\right)=\sigma_{b}\left(x,x\right). \end{array}$$

It is clear that the limit of a sequence in a *b*-metric-like space is usually not unique (since already partial metric spaces share this property).

We start our work by proving the following crucial lemma.

**Lemma 16**

Let (*X*, *σ*_*b*_) be a *b*-metric-like space with coefficient *s* > 1, and suppose that {*x*_*n*_} and {*y*_*n*_} are convergent to *x* and *y*, respectively. Then one has

(8) 1s2σb(x,y)−1sσb(x,x)−σb(y,y) ≤liminf⁡n→∞ σb(xn,yn)≤limsup⁡n→∞ σb(xn,yn) ≤sσb(x,x)+s2σb(y,y)+s2σb(x,y).

In particular, if *σ*_*b*_(*x*, *y*) = 0, then one has lim⁡_*n*→*∞*_⁡*σ*_*b*_(*x*_*n*_, *y*_*n*_) = 0.

Moreover, for each *z* ∈ *X* one has

(9) 1sσb(x,z)−σb(x,x) ≤liminf⁡n→∞ σb(xn,z)≤limsup⁡n→∞ σb(xn,z) ≤sσb(x,z)+sσb(x,x).

In particular, if *σ*_*b*_(*x*, *x*) = 0, then

(10) 1sσb(x,z)≤liminf⁡n→∞ σb(xn,z)≤limsup⁡n→∞ σb(xn,z)≤sσb(x,z).

**Proof**

Using the triangle inequality in a *b*-metric-like space it is easy to see that

(11) $$\begin{array}{c} \sigma_{b}\left(x,y\right)\leq s\sigma_{b}\left(x,x_{n}\right)+s^{2}\sigma_{b}\left(x_{n},y_{n}\right)+s^{2}\sigma_{b}\left(y_{n},y\right),\\ \sigma_{b}\left(x_{n},y_{n}\right)\leq s\sigma_{b}\left(x_{n},x\right)+s^{2}\sigma_{b}\left(x,y\right)+s^{2}\sigma_{b}\left(y,y_{n}\right). \end{array}$$

Taking the lower limit as *n* → *∞* in the first inequality and the upper limit as *n* → *∞* in the second inequality we obtain the first desired result. If *σ*_*b*_(*x*, *y*) = 0, then by the triangle inequality we get *σ*_*b*_(*x*, *x*) = 0 and *σ*_*b*_(*y*, *y*) = 0. Therefore, we have lim⁡_*n*→*∞*_ *σ*_*b*_(*x*_*n*_, *y*_*n*_) = 0. Similarly, using again the triangle inequality the other assertions follow.

### 2.2. Contraction Conditions and Fixed Point Results

It is well known that a self-map *f* on a metric space (*X*, *d*) is said to be a Banach contraction mapping, if there exists a number *k* ∈ [0,1) such that

(12) $$\begin{array}{c} d\left(fx,fy\right)\leq kd\left(x,y\right) \end{array}$$

for all *x*, *y* ∈ *X*. A mapping *f* : *X* → *X* is called a quasicontraction if for some constant *α* ∈ [0,1) and for every *x*, *y* ∈ *X*

(13) d(fx,fy)≤αmax⁡{d(x,y),d(x,fx),d(y,fy),d(x,fy),d(y,fx)}.

This concept was introduced and studied by Ćirić in 1974 [16]. A result of Ćirić shows that every quasicontraction in a complete metric space *f* has a unique fixed point.

The existence of fixed points in partially ordered metric spaces was first investigated in 2004 by Ran and Reurings [17] and then by Nieto and Rodríguez-López [18].

In this paper, we establish some fixed point theorems for quasicontractive type mappings in a partially ordered complete *b*-metric-like space. We investigate also the so-called *P*-property for mappings in such spaces.

## 3. Main Results

### 3.1. Fixed Points of Quasicontraction-Type Mappings

Throughout this paper, let (*X*, ⪯) be a partially ordered set, and let (*X*, *σ*_*b*_) be a *b*-metric-like space (we will say, for short, that (*X*, ⪯, *σ*_*b*_) is a partially ordered *b*-metric-like space). Further, let *F*(*f*) = {*x* ∈ *X* : *fx* = *x*} be the fixed point set of *f*, (*LF*)_*f*_ = {*x* ∈ *X* : *x*⪯*fx*} the lower fixed point set of *f*, and

(14) M(x,y)=max⁡{σb(x,y),σb(x,fx),σb(y,fy),σb(x,fy),σb(y,fx)}.

In this section, we obtain some fixed point results for quasicontractions defined on a partially ordered complete *b*-metric-like space.

We will also make use of the following notion.

**Definition 17**

An ordered *b*-metric-like space (*X*, ⪯, *σ*_*b*_) is said to have the sequential limit comparison property if for every nondecreasing sequence (nonincreasing sequence) {*x*_*n*_}_*n*∈*ℕ*_ in *X*, *x*_*n*_ → *x* implies that *x*_*n*_⪯*x*  (*x*⪯*x*_*n*_), for all *n* ∈ *ℕ*.

**Theorem 18**

Let (*X*, ⪯, *σ*_*b*_) be a complete partially ordered *b*-metric-like space. If *f* : *X* → *X* is a nondecreasing map such that, for all elements *x*, *y* ∈ *X* with *x*⪯*y*,

(15) $$\begin{array}{c} \sigma_{b}\left(fx,fy\right)\leq \alpha M\left(x,y\right), \end{array}$$

where *α* ∈ [0, 1/2*s* ^2^), then *F*(*f*) ≠ *∅* provided that there exists an *x*_0_ ∈ (*LF*)_*f*_ and one of the following two conditions is satisfied:

- *f* is a continuous self-map on *X*,
- (*X*, ⪯, *σ*_*b*_) has the sequential limit comparison property.

Moreover, *f* has a unique fixed point if and only if each pair of fixed points of *f* is comparable.

**Proof**

Since *x*_0_ ∈ (*LF*)_*f*_ and *f* is nondecreasing, therefore *f* ^*n*^ *x*_0_⪯*f* ^*n*+1^ *x*_0_ for each *n* ∈ *ℕ*. Define a sequence {*x*_*n*_} in *X* with *x*_*n*_ = *f* ^*n*^ *x*_0_ and so *x*_*n*+1_ = *fx*_*n*_ for all *n* ∈ *ℕ*. If there exists a positive integer *n* such that *x*_*n*_ = *x*_*n*+1_, then *f* ^*n*^ *x*_0_ = *f* ^*n*+1^ *x*_0_ = *ff* ^*n*^ *x*_0_ which implies that *f* ^*n*^ *x*_0_ is a fixed point of *f*. Assume that *x*_*n*_ ≠ *x*_*n*+1_ for every positive integer *n*. Since *x*_*n*−1_⪯*x*_*n*_, therefore by replacing *x* by *x*_*n*−1_ and *y* by *x*_*n*_ in (15), we have

(16) σb(xn,xn+1) =σb(fxn−1,fxn) ≤αmax⁡{σb(xn−1,xn),σb(xn−1,fxn−1),σb(xn,fxn), σb(xn−1,fxn),σb(xn,fxn−1)} =αmax⁡{σb(xn−1,xn),σb(xn−1,xn),σb(xn,xn+1), σb(xn−1,xn+1),σb(xn,xn)} ≤αmax⁡{σb(xn−1,xn),σb(xn,xn+1),sσb(xn−1,xn) +sσb(xn,xn+1),2sσb(xn−1,xn)}.

If, for some *n*, *σ*_*b*_(*x*_*n*_, *x*_*n*+1_) > *σ*_*b*_(*x*_*n*−1_, *x*_*n*_) > 0, then according to the above inequality *σ*_*b*_(*x*_*n*_, *x*_*n*+1_) ≤ 2*sασ*_*b*_(*x*_*n*_, *x*_*n*+1_) < *σ*_*b*_(*x*_*n*_, *x*_*n*+1_) which is a contradiction.

Hence, for all *n*, *σ*_*b*_(*x*_*n*_, *x*_*n*+1_) ≤ *σ*_*b*_(*x*_*n*−1_, *x*_*n*_) and therefore

(17) $$\begin{array}{c} \sigma_{b}\left(x_{n+1},x_{n}\right)\leq h\sigma_{b}\left(x_{n},x_{n-1}\right), \end{array}$$

where *h* = 2*αs*. Obviously, 0 ≤ *h* < 1. Repeating the above process, we get

(18) $$\begin{array}{c} \sigma_{b}\left(x_{n+1},x_{n}\right)\leq h\sigma_{b}\left(x_{n},x_{n-1}\right)\leq \cdots \leq h^{n}\sigma_{b}\left(x_{1},x_{0}\right), \end{array}$$

for all *n* ≥ 1, and so, for *m* > *n*, we have

(19) σb(xn,xm)≤sσb(xn,xn+1)+s2σb(xn+1,xn+2) +⋯+sm−nσb(xm−1,xm)≤shnσb(x0,x1)+s2hn+1σb(x0,x1) +⋯+sm−nhm−1σb(x0,x1)=shn(1+sh+⋯+sm−n−1hm−n−1)σb(x0,x1)≤shn1−shσb(x0,x1).

Since, by assumption, *h* < 1/*s*, it follows that lim⁡_*m*,*n*→*∞*_⁡*σ*_*b*_(*x*_*n*_, *x*_*m*_) = 0. Since *X* is complete, there exists an element *u* ∈ *X* such that

(20) $$\begin{array}{c} 0=\underset{n,m\to \infty }{\lim}\sigma_{b}\left(x_{n},x_{m}\right)=\underset{n\to \infty }{\lim}\sigma_{b}\left(x_{n},u\right)=\sigma_{b}\left(u,u\right). \end{array}$$

If *f* is a continuous self-map on *X*, then *fu* = *u*. Indeed, by the triangle inequality we have

(21) σb(fu,u)≤s(σb(fu,xn+1)+σb(xn+1,u))=s(σb(fu,fxn)+σb(xn+1,u)).

Taking the limit as *n* → *∞* in the above inequality, the desired result is obtained.

If condition (b) is fulfilled then *x*_*n*_ = *f* ^*n*^ *x*_0_⪯*u* for all *n* ∈ *ℕ*, and it follows that

(22) σb(fxn,fu)≤αmax⁡{σb(xn,u),σb(xn,fxn),σb(u,fu),σb(xn,fu),σb(u,fxn)}=αmax⁡{σb(xn,u),σb(xn,xn+1),σb(u,fu),σb(xn,fu),σb(u,xn+1)}.

On taking the upper limit as *n* → *∞*, (using Lemma 16, as *σ*_*b*_(*u*, *u*) = 0) it follows that

(23) $$\begin{array}{c} \frac{1}{s}\sigma_{b}\left(u,fu\right)\leq \alpha s\sigma_{b}\left(u,fu\right), \end{array}$$

and hence *σ*_*b*_(*u*, *fu*) ≤ *αs* ^2^ *σ*_*b*_(*u*, *fu*), or equivalently, *fu* = *u*.

Suppose that the fixed points of *f* are comparable. Let *w* be another fixed point of *f* such that *w* ≠ *u*. Assume, for example, that *u*⪯*w*. Using (15), we obtain that

(24) σb(u,w)=σb(fu,fw)≤αmax⁡{σb(u,w),σb(u,fu),σb(w,fw),σb(u,fw),σb(w,fu)}=αmax⁡⁡{σb(u,w),σb(u,u),σb(w,w),σb(u,w),σb(w,u)}≤2sασb(u,w),

which further implies that *u* = *w*. The converse is trivial.

**Example 19**

Let *X* = [0,1] be endowed with the usual order. Define *σ*_*b*_ : *X* × *X* → ℝ^+^ by

(25) $$\begin{array}{c} \sigma_{b}\left(x,y\right)=x^{2}+y^{2}+{\left|x-y\right|}^{2} \end{array}$$

for all *x*, *y* ∈ *X*. Then (*X*, *σ*_*b*_) is a complete *b*-metric-like space with coefficient *s* = 2 (see Example 14).

Let *f* : *X* → *X* be defined by *fx* = ln⁡(1 + *x*/4). It is easy to see that *f* is a nondecreasing and continuous self-map on *X*, and 0 ∈ (*LF*)_*f*_. Using the Mean Value Theorem for any *x*, *y* ∈ *X* with *x* ≤ *y* and that *fx* ≤ *x*/4, we have

(26) σb(fx,fy)=f2x+f2y+|fy−fx|2=(ln⁡(1+x4))2+(ln⁡(1+y4))2 +|ln⁡(1+y4)−ln⁡(1+x4)|2≤(x4)2+(y4)2+|y4−x4|2=116(x2+y2+|x−y|2)=116σb(x,y)≤116M(x,y).

Thus (15) is satisfied with *α* = 1/16 ∈ [0, 1/8). Thus all conditions of Theorem 18 are satisfied. Moreover, 0 is the unique fixed point of *f*.

**Theorem 20**

Let (*X*, ⪯, *σ*_*b*_) be a partially ordered complete *b*-metric-like space with coefficient *s* > 1. If a nondecreasing map *f* : *X* → *X* satisfies

(27) $$\begin{array}{c} s\sigma_{b}\left(fx,fy\right)\leq M\left(x,y\right)-\varphi \left(m\left(x,y\right)\right), \end{array}$$

for all *x*, *y* ∈ *X* with *x*⪯*y*, where

(28) M(x,y)=σb(x,fy)+σb(y,fx)4s,m(x,y)=min⁡{σb(x,fy),σb(y,fx)},

and *φ* : [0, *∞*) → [0, *∞*) is a continuous and nondecreasing function such that *φ*(*t*) > 0 for all *t* ∈ (0, *∞*) and *φ*(0) = 0, then *F*(*f*) ≠ *∅* provided that there exists an *x*_0_ ∈ (*LF*)_*f*_ and one of the following two conditions is satisfied:

- *f* is a continuous self-map on *X*.
- (*X*, ⪯, *σ*_*b*_) has the sequential limit comparison property.

Moreover, *f* has a unique fixed point if and only if the fixed points of *f* are comparable.

**Proof**

Define the sequence {*x*_*n*_} as given in the proof of Theorem 18. If there exists a positive integer *n* such that *x*_*n*_ = *x*_*n*+1_, then *x*_*n*_ is a fixed point of *f*. Assume that *x*_*n*_ ≠ *x*_*n*+1_ for every positive integer *n*. Since *x*_*n*−1_⪯*x*_*n*_, therefore by replacing *x* by *x*_*n*−1_ and *y* by *x*_*n*_ in (27), we obtain

(29) sσb(xn,xn+1) =sσb(fxn−1,fxn) ≤M(xn−1,xn)−φ(m(xn−1,xn)) ≤σb(xn−1,fxn)+σb(xn,fxn−1)4s−φ(m(xn−1,xn)) =σb(xn−1,xn+1)+σb(xn,xn)4s−φ(m(xn−1,xn)) ≤sσb(xn−1,xn)+sσb(xn,xn+1)+2sσb(xn,xn+1)4s  −φ(m(xn−1,xn)) ≤sσb(xn−1,xn)+sσb(xn,xn+1)+2sσb(xn,xn+1)4s.

Therefore, for all *n* ≥ 0, *σ*_*b*_(*x*_*n*_, *x*_*n*+1_) ≤ *σ*_*b*_(*x*_*n*_, *x*_*n*−1_) and {*σ*_*b*_(*x*_*n*_, *x*_*n*+1_)} is nonincreasing and bounded from below. Hence, there exists *r* ≥ 0 such that lim⁡_*n*→*∞*_⁡*σ*_*b*_(*x*_*n*+1_, *x*_*n*_) = *r*.

From the above argument we have

(30) sσb(xn+1,xn) ≤σb(xn−1,xn+1)+σb(xn,xn)4s ≤sσb(xn−1,xn)+sσb(xn,xn+1)+2sσb(xn,xn+1)4s.

If *n* → *∞*, we get

(31) $$\begin{array}{c} sr\leq \underset{n\to \infty }{\lim}\frac{\sigma_{b}\left(x_{n-1},x_{n+1}\right)+\sigma_{b}\left(x_{n},x_{n}\right)}{4s}\leq r, \end{array}$$

which is, because of *s* > 1, possible only if *r* = 0. So we have

(32) $$\begin{array}{c} \underset{n\to \infty }{\lim}\sigma_{b}\left(x_{n+1},x_{n}\right)=0. \end{array}$$

Next, we show that {*x*_*n*_} is a Cauchy sequence. If not, then there exists *ɛ* > 0 for which we can find subsequences {*x*_*m*_*k*__} and {*x*_*n*_*k*__} of the sequence {*x*_*n*_} where *n*_*k*_ is the smallest index for which *n*_*k*_ > *m*_*k*_ > *k* with

(33) $$\begin{array}{c} \sigma_{b}\left(x_{m_{k}},x_{n_{k}}\right)\geq ɛ. \end{array}$$

Then,

(34) $$\begin{array}{c} \sigma_{b}\left(x_{m_{k}},x_{n_{k}-1}\right)<ɛ. \end{array}$$

From (33) and (34), we obtain

(35) ɛ≤σb(xmk,xnk)≤sσb(xmk,xnk−1)+sσb(xnk−1,xnk)<sɛ+sσb(xnk−1,xnk).

Taking the upper and lower limits as *k* → *∞*, from (32) we conclude that

(36) $$\begin{array}{c} ɛ\leq \underset{k\to \infty }{\mathrm{liminf}}\;\sigma_{b}\left(x_{m_{k}},x_{n_{k}}\right)\leq \underset{k\to \infty }{\mathrm{limsup}}\;\sigma_{b}\left(x_{m_{k}},x_{n_{k}}\right)\leq sɛ. \end{array}$$

Note that

(37) $$\begin{array}{c} \sigma_{b}\left(x_{m_{k}+1},x_{n_{k}-1}\right)\leq s\sigma_{b}\left(x_{m_{k}+1},x_{m_{k}}\right)+s\sigma_{b}\left(x_{m_{k}},x_{n_{k}-1}\right). \end{array}$$

Using (34) and (32), we get

(38) $$\begin{array}{c} \underset{k\to \infty }{\mathrm{limsup}}\;\sigma_{b}\left(x_{m_{k}+1},x_{n_{k}-1}\right)\leq sɛ. \end{array}$$

On the other hand,

(39) σb(xmk,xnk)≤sσb(xmk,xmk+1)+s2σb(xmk+1,xnk−1) +s2σb(xnk−1,xnk).

Using (36) and (32), we get

(40) $$\begin{array}{c} \frac{ɛ}{s^{2}}\leq \underset{k\to \infty }{\mathrm{liminf}}\;\sigma_{b}\left(x_{m_{k}+1},x_{n_{k}-1}\right). \end{array}$$

From (38) and (40), we have

(41) ɛs2≤liminf⁡k→∞ σb(xmk+1,xnk−1)≤limsup⁡k→∞ σb(xmk+1,xnk−1)≤sɛ.

Also,

(42) $$\begin{array}{c} ɛ\leq \sigma_{b}\left(x_{m_{k}},x_{n_{k}}\right)\leq s\sigma_{b}\left(x_{m_{k}},x_{m_{k}+1}\right)+s\sigma_{b}\left(x_{m_{k}+1},x_{n_{k}}\right). \end{array}$$

Taking the upper limit as *k* → *∞*, we conclude that

(43) $$\begin{array}{c} ɛ\leq \underset{k\to \infty }{\mathrm{limsup}}\;\sigma_{b}\left(x_{m_{k}},x_{n_{k}}\right)\leq \underset{k\to \infty }{\mathrm{limsup}}\;s\sigma_{b}\left(x_{m_{k}+1},x_{n_{k}}\right), \end{array}$$

or equivalently,

(44) $$\begin{array}{c} \frac{ɛ}{s}\leq \underset{k\to \infty }{\mathrm{limsup}}\;\sigma_{b}\left(x_{m_{k}+1},x_{n_{k}}\right). \end{array}$$

As {*x*_*n*_} is nondecreasing and *m*_*k*_ < *n*_*k*_, from (27) we have

(45) sσb(xmk+1,xnk)=sσb(fxmk,fxnk−1)≤M(xmk,xnk−1)−φ(m(xmk,xnk−1)),

where

(46) M(xmk,xnk−1)=σb(xmk,fxnk−1)+σb(xnk−1,fxmk)4s=σb(xmk,xnk)+σb(xnk−1,xmk+1)4s,m(xmk,xnk−1)=min⁡{σb(xmk,xnk),σb(xnk−1,xmk+1)},

wherefrom on taking the upper limit as *k* → *∞*, from (36) and (41), we have

(47) $$\begin{array}{c} \underset{k\to \infty }{\mathrm{limsup}}\;M\left(x_{m_{k}},x_{n_{k}-1}\right)\leq \frac{ɛ}{2},\\ \underset{k\to \infty }{\mathrm{liminf}}\;m\left(x_{m_{k}},x_{n_{k}-1}\right)\geq \min\left\{ɛ,\frac{ɛ}{s^{2}}\right\}=\frac{ɛ}{s^{2}}. \end{array}$$

Thus, from (44) and (45), we have

(48) $$\begin{array}{c} ɛ\leq \frac{ɛ}{2}-\varphi \left(\frac{ɛ}{s^{2}}\right), \end{array}$$

which gives *ɛ* = 0, a contradiction. Hence {*x*_*n*_} is a Cauchy sequence in *X*. Since *X* is complete, there exists an element *u* ∈ *X* such that

(49) $$\begin{array}{c} 0=\underset{n,m\to \infty }{\lim}\sigma_{b}\left(x_{n},x_{m}\right)=\underset{n\to \infty }{\lim}\sigma_{b}\left(x_{n},u\right)=\sigma_{b}\left(u,u\right). \end{array}$$

If *f* is a continuous self-map on *X*, then *fu* = *u*. If assumption (b) is satisfied then *x*_*n*_ = *f* ^*n*^ *x*_0_⪯*u* for all *n* ∈ *ℕ*; it follows that

(50) $$\begin{array}{c} s\sigma_{b}\left(fx_{n},fu\right)\leq M\left(x_{n},u\right)-\varphi \left(m\left(x_{n},u\right)\right), \end{array}$$

where

(51) $$\begin{array}{c} M\left(x_{n},u\right)=\frac{\sigma_{b}\left(x_{n},fu\right)+\sigma_{b}\left(u,x_{n+1}\right)}{4s},\\ m\left(x_{n},u\right)=\min\left\{\sigma_{b}\left(x_{n},fu\right),\sigma_{b}\left(u,x_{n+1}\right)\right\}. \end{array}$$

On taking the upper limit as *n* → *∞* in (50), from Lemma 16 we obtain

(52) $$\begin{array}{c} s\cdot \frac{1}{s}\sigma_{b}\left(u,fu\right)\leq \frac{s\sigma_{b}\left(u,fu\right)}{4s}-\varphi \left(0\right), \end{array}$$

and hence *σ*_*b*_(*u*, *fu*) = 0, implying that *u* = *fu*.

Now, suppose that the fixed points of *f* are comparable. Let *w* be another fixed point of *f*. We will show that *w* = *u*. If not, then without loss of generality, we assume that *u*⪯*w*. Using (27), we obtain

(53) $$\begin{array}{c} \sigma_{b}\left(u,w\right)=\sigma_{b}\left(fu,fw\right)\leq M\left(u,w\right)-\varphi \left(m\left(u,w\right)\right), \end{array}$$

where

(54) M(u,w)=σb(u,fw)+σb(w,fu)4s=σb(u,w)+σb(w,u)4s=σb(u,w)2s.

Thus,

(55) σb(u,w)=σb(fu,fw)≤σb(u,w)2s−φ(σb(u,w)),

which implies that *σ*_*b*_(*u*, *w*) = 0, which yields that *u* = *w*. The converse is trivial.

**Example 21**

Let *X* = [0, *∞*) be endowed with the usual order ≤. Define *σ*_*b*_ : *X* × *X* → ℝ^+^ by

(56) $$\begin{array}{c} \sigma_{b}\left(x,y\right)={\left(x^{2}+y^{2}\right)}^{2} \end{array}$$

for all *x*, *y* ∈ *X*. Then (*X*, ≤, *σ*_*b*_) is a complete ordered *b*-metric-like space with coefficient *s* = 2 (see Example 11).

Let *f* : *X* → *X* be defined by $fx=\left(1/4\right)\sqrt{\ln\left(\left(x^{2}/3\right)+1\right)}$. It is easy to see that *f* is a nondecreasing and continuous self-map on *X*, and 0 ∈ (*LF*)_*f*_. Using the Mean Value Theorem for the function *f* ^2^ *x* = (1/16)ln⁡(*x* ^2^/3 + 1) for any *x*, *y* ∈ *X* with *x* ≤ *y*, we have

(57) f2y−f2x=116(ln⁡(y23+1)−ln⁡(x23+1))≤148(y2−x2)≤y2−x2,

which implies that

(58) $$\begin{array}{c} f^{2}y+x^{2}\leq f^{2}x+y^{2}. \end{array}$$

Hence

(59) $$\begin{array}{c} m\left(x,y\right)=\min\left\{\sigma_{b}\left(fy,x\right),\sigma_{b}\left(fx,y\right)\right\}=\sigma_{b}\left(fy,x\right). \end{array}$$

Now we define *φ* : [0, *∞*) → [0, *∞*) by *φ*(*t*) = *t*/8, and, for any *x*, *y* ∈ *X* with *x* ≤ *y*, we have

(60) sσb(fx,fy)≤M(x,y)−φ(m(x,y))⟺2σb(fx,fy)≤σb(fy,x)+σb(fx,y)8  −σb(fy,x)8⟺16σb(fx,fy)≤σb(fx,y)⟺16(f2x+f2y)2≤(f2x+y2)2⟺4(f2x+f2y)≤f2x+y2⟺3f2x+4f2y≤y2⟸7f2y≤y2⟺f2y≤y27⟸116ln⁡(y23+1)≤y248,

since *f* ^2^ *x* = (1/16)ln⁡(*x* ^2^/3 + 1) is an increasing function and *f* ^2^ *x* ≤ *x* ^2^/48.

Thus, all conditions of Theorem 20 are satisfied. Moreover, 0 is the unique fixed point of *f*.

### 3.2. Coincidence Points of Four Mappings under Generalized Weakly Contractive Conditions

**Definition 22**

Let *f* and *g* be two self-maps on partially ordered set *X*. A pair (*f*, *g*) is said to be

- weakly increasing if *fx*⪯*g* *fx* and *gx*⪯*f* *gx*, for all *x* ∈ *X* [19, 20],
- partially weakly increasing if *fx*⪯*g* *fx*, for all *x* ∈ *X* [21].

Let *X* be a nonempty set and *f* : *X* → *X* a given mapping. For every *x* ∈ *X*, let *f* ^−1^(*x*) = {*u* ∈ *X* : *fu* = *x*}.

**Definition 23**

Let (*X*, ⪯) be a partially ordered set and *f*, *g*, *h* : *X* → *X* mappings such that *fX*⊆*hX* and *gX*⊆*hX*. The ordered pair (*f*, *g*) is said to be

- weakly increasing with respect to *h* if and only if, for all *x* ∈ *X*, *fx*⪯*gy* for all *y* ∈ *h* ^−1^(*fx*), and *gx*⪯*fy* for all *y* ∈ *h* ^−1^(*gx*) [22],
- partially weakly increasing with respect to *h* if *fx*⪯*gy*, for all *y* ∈ *h* ^−1^(*fx*) [23].

**Remark 24**

In the above definition (i) if *f* = *g*, we say that *f* is weakly increasing (partially weakly increasing) with respect to *h* and (ii) if *h* = *I* (the identity mapping on *X*), then the above definition reduces to the weakly increasing (partially weakly increasing) mapping (see [22, 24]).

The study of unique common fixed points of mappings satisfying weakly contractive conditions has been at the center of vigorous research activity. Motivated by the work in [21, 23–30], we prove some coincidence point results for nonlinear generalized (*ψ*, *φ*)-weakly contractive mappings in partially ordered *b*-metric-like spaces.

Recall [31] that an altering distance function is a mapping *ψ* : [0, *∞*) → [0, *∞*) which satisfies that

(*ψ*_1_) *ψ*(*t*) is increasing and continuous,
(*ψ*_2_) *ψ*(*t*) = 0 if and only if *t* = 0.

Let (*X*, ⪯, *σ*_*b*_) be an ordered *b*-metric-like space and *f*, *g*, *R*, *S* : *X* → *X* four self-mappings. Throughout this subsection, unless otherwise stated, for all *x*, *y* ∈ *X*, let

(61) M(x,y)=max⁡{σb(Sx,Ry),σb(Sx,fx),σb(Ry,gy),σb(Sx,gy)+σb(Ry,fx)4s}.

Suppose that *f*(*X*)⊆*R*(*X*) and *g*(*X*)⊆*S*(*X*); let *x*_0_ be an arbitrary point of *X*. Choose *x*_1_ ∈ *X* such that *fx*_0_ = *Rx*_1_ and *x*_2_ ∈ *X* such that *gx*_1_ = *Sx*_2_. Continuing in this way, construct a sequence {*z*_*n*_} defined by *z*_2*n*+1_ = *Rx*_2*n*+1_ = *fx*_2*n*_ and *z*_2*n*+2_ = *Sx*_2*n*+2_ = *gx*_2*n*+1_, for all *n* ≥ 0. The sequence {*z*_*n*_} in *X* is said to be a Jungck-type iterative sequence with initial guess *x*_0_.

**Theorem 25**

Let (*X*, ⪯, *σ*_*b*_) be a partially ordered *b*-metric-like space with sequential limit comparison property, *f*, *g*, *R*, *S* : *X* → *X* four mappings such that *f*(*X*)⊆*R*(*X*) and *g*(*X*)⊆*S*(*X*), and *RX* and *SXσ*_*b*_-complete subsets of *X*. Suppose that, for comparable elements *Sx*, *Ry* ∈ *X*, one has

(62) $$\begin{array}{c} \psi \left(s^{2}\sigma_{b}\left(fx,gy\right)\right)\leq \psi \left(M\left(x,y\right)\right)-\varphi \left(M\left(x,y\right)\right), \end{array}$$

where *ψ*, *φ* : [0, *∞*) → [0, *∞*) are altering distance functions. Then, the pairs (*f*, *S*) and (*g*, *R*) have a coincidence point z* in *X* provided that the pairs (*f*, *S*) and (*g*, *R*) are weakly compatible, and the pairs (*f*, *g*) and (*g*, *f*) are partially weakly increasing with respect to *R* and *S*, respectively.

**Proof**

Let {*z*_*n*_} be a Jungck-type iterative sequence with initial guess *x*_0_ in *X*; that is, *z*_2*n*+1_ = *Rx*_2*n*+1_ = *fx*_2*n*_, *z*_2*n*+2_ = *Sx*_2*n*+2_ = *gx*_2*n*+1_ for all *n* ≥ 0.

As *x*_1_ ∈ *R* ^−1^(*fx*_0_) and *x*_2_ ∈ *S* ^−1^(*gx*_1_), and the pairs (*f*, *g*) and (*g*, *f*) are partially weakly increasing with respect to *R* and *S*, so we have

(63) $$\begin{array}{c} Rx_{1}=fx_{0}⪯gx_{1}=Sx_{2}. \end{array}$$

Continuing this process, we obtain *Rx*_2*n*+1_⪯*Sx*_2*n*+2_, for *n* ≥ 0. We will complete the proof in three steps.

*Step  I.* We will prove that lim⁡_*k*→*∞*_⁡*σ*_*b*_(*z*_*k*_, *z*_*k*+1_) = 0.

Define *σ*_*b*_*k*__ = *σ*_*b*_(*z*_*k*_, *z*_*k*+1_). Suppose *σ*_*b*_*k*_0___ = 0, for some *k*_0_. Then, *z*_*k*_0__ = *z*_*k*_0_+1_. If *k*_0_ = 2*n*, then *z*_2*n*_ = *z*_2*n*+1_ gives *z*_2*n*+1_ = *z*_2*n*+2_. Indeed,

(64) ψ(σb(z2n+1,z2n+2))=ψ(σb(fx2n,gx2n+1))≤ψ(s2σb(fx2n,gx2n+1))≤ψ(M(x2n,x2n+1)) −φ(M(x2n,x2n+1)),

where

(65) M(x2n,x2n+1) =max⁡{σb(Sx2n,Rx2n+1),σb(Sx2n,fx2n),σb(Rx2n+1,gx2n+1),σb(Sx2n,gx2n+1)+σb(Rx2n+1,fx2n)4s} =max⁡{σb(z2n,z2n+1),σb(z2n,z2n+1),σb(z2n+1,z2n+2),σb(z2n,z2n+2)+σb(z2n+1,z2n+1)4s} ≤max⁡{σb(z2n,z2n+1),σb(z2n,z2n+1),σb(z2n+1,z2n+2),(sσb(z2n,z2n+1)+sσb(z2n+1,z2n+2)+2sσb(z2n+1,z2n+2))×(4s)−1} =max⁡{0,0,σb(z2n+1,z2n+2),0+3sσb(z2n+1,z2n+2)4s} =max⁡{σb(z2n+1,z2n+2),3σb(z2n+1,z2n+2)4} =σb(z2n+1,z2n+2).

Thus,

(66) ψ(σb(z2n+1,z2n+2))≤ψ(σb(z2n+1,z2n+2)) −φ(M(z2n,z2n+1)),

which implies that *φ*(*M*(*z*_2*n*_, *z*_2*n*+1_)) = 0; that is, *z*_2*n*+1_ = *z*_2*n*+2_. Similarly, if *k*_0_ = 2*n* + 1, then *z*_2*n*+1_ = *z*_2*n*+2_ gives *z*_2*n*+2_ = *z*_2*n*+3_. Consequently, the sequence {*z*_*k*_} becomes constant for *k* ≥ *k*_0_ and hence lim⁡_*k*→*∞*_⁡*σ*_*b*_(*z*_*k*_, *z*_*k*+1_) = 0.

Suppose that

(67) $$\begin{array}{c} \sigma_{b_{k}}=\sigma_{b}\left(z_{k},z_{k+1}\right)>0, \end{array}$$

for each *k*. We now claim that the following inequality holds:

(68) $$\begin{array}{c} \sigma_{b}\left(z_{k+1},z_{k+2}\right)\leq \sigma_{b}\left(z_{k},z_{k+1}\right)=M\left(x_{k},x_{k+1}\right) \end{array}$$

for each *k* = 1,2,….

Let *k* = 2*n*, and for an *n* ≥ 0, *σ*_*b*_(*z*_2*n*+1_, *z*_2*n*+2_) > *σ*_*b*_(*z*_2*n*_, *z*_2*n*+1_) > 0. Then, as *Sx*_2*n*_⪯*Rx*_2*n*+1_, using (62) we obtain that

(69) ψ(σb(z2n+1,z2n+2)) ≤ψ(s2σb(z2n+1,z2n+2)) =ψ(s2σb(fx2n,gx2n+1)) ≤ψ(M(x2n,x2n+1))−φ(M(x2n,x2n+1)),

where

(70) M(x2n,x2n+1) =max⁡{σb(Sx2n,Rx2n+1),σb(Sx2n,fx2n),σb(Rx2n+1,gx2n+1),σb(Sx2n,gx2n+1)+σb(Rx2n+1,fx2n)4s} =max⁡{σb(z2n,z2n+1),σb(z2n,z2n+1),σb(z2n+1,z2n+2),σb(z2n,z2n+2)+σb(z2n+1,z2n+1)4s} ≤max⁡{σb(z2n,z2n+1),σb(z2n+1,z2n+2),(sσb(z2n,z2n+1)+sσb(z2n+1,z2n+2)+2sσb(z2n+1,z2n+2))×(4s)−1} =max⁡{σb(z2n,z2n+1),σb(z2n+1,z2n+2),sσb(z2n,z2n+1)+3sσb(z2n+1,z2n+2)4s} =max⁡{σb(z2n,z2n+1),σb(z2n+1,z2n+2)}.

If, for some *n*, *σ*_*b*_(*z*_2*n*+1_, *z*_2*n*+2_) > *σ*_*b*_(*z*_2*n*_, *z*_2*n*+1_) > 0, (69) implies that

(71) ψ(σb(z2n+1,z2n+2))≤ψ(σb(z2n+1,z2n+2)) −φ(M(x2n,x2n+1)),

which is possible only if *M*(*x*_2*n*_, *x*_2*n*+1_) = 0; that is, *σ*_*b*_(*z*_2*n*_, *z*_2*n*+1_) = 0, a contradiction to (67). Hence, *σ*_*b*_(*z*_2*n*+1_, *z*_2*n*+2_) ≤ *σ*_*b*_(*z*_2*n*_, *z*_2*n*+1_) and

(72) $$\begin{array}{c} M\left(x_{2n},x_{2n+1}\right)=\sigma_{b}\left(z_{2n},z_{2n+1}\right). \end{array}$$

Therefore, (68) is proved for *k* = 2*n*.

Similarly, it can be shown that

(73) $$\begin{array}{c} \sigma_{b}\left(z_{2n+2},z_{2n+3}\right)\leq \sigma_{b}\left(z_{2n+1},z_{2n+2}\right)=M\left(x_{2n+1},x_{2n+2}\right). \end{array}$$

Hence, {*σ*_*b*_(*z*_*k*_, *z*_*k*+1_)} is a nonincreasing sequence of nonnegative real numbers. Therefore, there is an *r* ≥ 0 such that

(74) $$\begin{array}{c} \underset{k\to \infty }{\lim}\sigma_{b}\left(z_{k},z_{k+1}\right)=r. \end{array}$$

Taking the limit as *k* → *∞* in (68), we obtain

(75) $$\begin{array}{c} \underset{k\to \infty }{\lim}M\left(x_{k},x_{k+1}\right)=r. \end{array}$$

Taking the limit as *n* → *∞* in (69), using (74), (75), and the continuity of *ψ* and *φ*, we have *ψ*(*r*) ≤ *ψ*(*r*) − *φ*(*r*). Therefore *φ*(*r*) = 0. Hence,

(76) $$\begin{array}{c} \underset{k\to \infty }{\lim}\sigma_{b}\left(z_{k},z_{k+1}\right)=0, \end{array}$$

from our assumptions about *φ*.

*Step  II.* We now show that {*z*_*n*_} is a *σ*_*b*_-Cauchy sequence in *X*. Because of (76), it is sufficient to show that {*z*_2*n*_} is *σ*_*b*_-Cauchy.

We assume on the contrary that there exists *ɛ* > 0 for which we can find subsequences {*z*_2*m*(*k*)_} and {*z*_2*n*(*k*)_} of {*z*_2*n*_} such that *n*(*k*) > *m*(*k*) ≥ *k* and

(77) $$\begin{array}{c} \sigma_{b}\left(z_{2m(k)},z_{2n(k)}\right)\geq ɛ, \end{array}$$

and *n*(*k*) is the smallest number such that the above statement holds; that is,

(78) $$\begin{array}{c} \sigma_{b}\left(z_{2m(k)},z_{2n(k)-2}\right)<ɛ. \end{array}$$

From triangle inequality, we have

(79) σb(z2m(k),z2n(k))≤sσb(z2m(k),z2m(k)+1) +sσb(z2m(k)+1,z2n(k)).

Taking the upper limit as *k* → *∞* in (79), from (77) and (76) we obtain that

(80) $$\begin{array}{c} \frac{ɛ}{s}\leq \underset{k\to \infty }{\mathrm{limsup}}\;\sigma_{b}\left(z_{2m(k)+1},z_{2n(k)}\right). \end{array}$$

Using triangle inequality, we have

(81) σb(z2m(k),z2n(k)−1)≤sσb(z2m(k),z2n(k)−2) +sσb(z2n(k)−2,z2n(k)−1).

Taking the upper limit as *k* → *∞* in (81), from (78) and (76) we have

(82) $$\begin{array}{c} \underset{k\to \infty }{\mathrm{limsup}}\;\sigma_{b}\left(z_{2m(k)},z_{2n(k)-1}\right)\leq sɛ. \end{array}$$

Also,

(83) σb(z2m(k),z2n(k))≤sσb(z2m(k),z2n(k)−2) +sσb(z2n(k)−2,z2n(k)).

Taking the upper limit as *k* → *∞* in (83) and using (76) and (78) we have

(84) $$\begin{array}{c} \underset{k\to \infty }{\mathrm{limsup}}\;\sigma_{b}\left(z_{2m(k)},z_{2n(k)}\right)\leq sɛ. \end{array}$$

Consider

(85) σb(z2m(k)+1,z2n(k)−1)≤sσb(z2m(k)+1,z2m(k)) +sσb(z2m(k),z2n(k)−1).

Taking the limit as *k* → *∞* and using (76) and (82), we have

(86) $$\begin{array}{c} \underset{k\to \infty }{\mathrm{limsup}}\;\sigma_{b}\left(z_{2m(k)+1},z_{2n(k)-1}\right)\leq s^{2}ɛ. \end{array}$$

As *Sx*_2*m*(*k*)_⪯*Rx*_2*n*(*k*)−1_, so from (62), we have

(87) ψ(σb(z2m(k)+1,z2n(k)))=ψ(σb(fx2m(k),gx2n(k)−1))≤ψ(M(x2m(k),x2n(k)−1)) −φ(M(x2m(k),x2n(k)−1)),

where

(88) M(x2m(k),x2n(k)−1) =max⁡{σb(Sx2m(k),Rx2n(k)−1),σb(Sx2m(k),fx2m(k)),σb(Rx2n(k)−1,gx2n(k)−1),σb(Sx2m(k),gx2n(k)−1)+σb(Rx2n(k)−1,fx2m(k))4s} =max⁡{σb(z2m(k),z2n(k)−1),σb(z2m(k),z2m(k)+1),σb(z2n(k)−1,z2n(k)),σb(z2m(k),z2n(k))+σb(z2n(k)−1,z2m(k)+1)4s}.

Taking the upper limit in the above and using (76), (82), (84), and (86), we get

(89) $$\begin{array}{c} \underset{k\to \infty }{\mathrm{limsup}}\;M\left(x_{2m(k)},x_{2n(k)-1}\right)\leq sɛ. \end{array}$$

Now, taking the upper limit as *k* → *∞* in (87) and using (80) and (89) we have

(90) ψ(sɛ)=ψ(s2ɛs)≤ψ(s2limsup⁡k→∞ σb(z2m(k)+1,z2n(k)))≤ψ(limsup⁡k→∞ M(x2m(k),x2n(k)−1)) −φ(liminf⁡k→∞ M(x2m(k),x2n(k)−1))≤ψ(sɛ)−φ(liminf⁡k→∞ M(x2m(k),x2n(k)−1)),

which implies that *φ*(liminf⁡_*k*→*∞*_⁡*M*(*x*_2*m*(*k*)_, *x*_2*n*(*k*)−1_)) = 0. Hence,

(91) $$\begin{array}{c} \underset{k\to \infty }{\mathrm{liminf}}\;\sigma_{b}\left(x_{2m(k)},x_{2n(k)}\right)=0, \end{array}$$

which is in contradiction with (77). Hence, {*z*_*n*_} is a *σ*_*b*_-Cauchy sequence.

*Step  III*. We will show that *f*, *g*, *R*, and *S* have a coincidence point.

Since {*z*_*n*_} is a *σ*_*b*_-Cauchy sequence and *R*(*X*) and *S*(*X*) are *σ*_*b*_-complete *σ*_*b*_-metric spaces, there exists z* ∈ *R*(*X*)∩*S*(*X*) such that

(92) $$\begin{array}{c} \underset{k\to \infty }{\lim}\sigma_{b}\left(z_{k},z^{*}\right)=\sigma_{b}\left(z^{*},z^{*}\right)=\underset{m,n\to \infty }{\lim}\sigma_{b}\left(z_{n},z_{m}\right)=0. \end{array}$$

There exists *u* ∈ *X* such that z* = *Ru* and

(93) $$\begin{array}{c} \underset{n\to \infty }{\lim}\sigma_{b}\left(z_{2n+1},Ru\right)=\underset{n\to \infty }{\lim}\sigma_{b}\left(Rx_{2n+1},Ru\right)=\sigma_{b}\left(Ru,Ru\right). \end{array}$$

Similarly, there exists *v* ∈ *X* such that z* = *Sv* and

(94) $$\begin{array}{c} \underset{n\to \infty }{\lim}\sigma_{b}\left(z_{2n+2},Sv\right)=\underset{n\to \infty }{\lim}\sigma_{b}\left(Sx_{2n+2},Sv\right)=\sigma_{b}\left(Sv,Sv\right). \end{array}$$

Now, we prove that z* is a coincidence point of *g* and *R*. For this purpose, we show that *Ru* = *gu*. Since *Sx*_2*n*+2_ → z* = *Ru* as *n* → *∞*, so *Sx*_2*n*+2_⪯*Ru*.

Therefore, from (62), we have

(95) ψ(s2σb(fx2n+2,gu))≤ψ(M(x2n+2,u)) −φ(M(x2n+2,u)),

where

(96) M(x2n+2,u)=max⁡{σb(Sx2n+2,Ru),σb(Sx2n+2,fx2n+2),σb(Ru,gu),σb(Sx2n+2,gu)+σb(Ru,fx2n+2)4s}.  

Taking the limit as *n* → *∞* in (95), as *σ*_*b*_(z*, z*) = 0, and using Lemma 16 we obtain that

(97) ψ(sσb(z∗,gu)) =ψ(s21sσb(z∗,gu)) ≤ψ(max⁡{sσb(z∗,Ru),0,σb(Ru,gu),σb(z∗,gu)+σb(z∗,Ru)4s})  −φ(max⁡{sσb(z∗,Ru),0,σb(Ru,gu),σb(z∗,gu)+σb(z∗,Ru)4s}) =ψ(σb(z∗,gu))−φ(σb(z∗,gu)) ≤ψ(sσb(z∗,gu))−φ(σb(z∗,gu))

which implies that *Ru* = z* = *gu*.

As *g* and *R* are weakly compatible, we have gz* = *g* *Ru* = *R* *gu* = Rz*. Thus z* is a coincidence point of *g* and *R*.

Similarly it can be shown that z* is a coincidence point of the pair (*f*, *S*)

**Theorem 26**

Let (*X*, ⪯, *σ*_*b*_) be a partially ordered *σ*_*b*_-complete *σ*_*b*_-metric-like space. Let *f*, *g* : *X* → *X* be two mappings. Suppose that for every two comparable elements *x*, *y* ∈ *X* one has

(98) $$\begin{array}{c} \psi \left(2s\sigma_{b}\left(fx,gy\right)\right)\leq \psi \left(M\left(x,y\right)\right)-\varphi \left(M\left(x,y\right)\right), \end{array}$$

where *ψ*, *φ* : [0, *∞*) → [0, *∞*) are altering distance functions and

(99) M(x,y)=max⁡{σb(x,y),σb(x,fx),σb(y,gy),σb(x,gy)+σb(y,fx)4s}.

Let *f* and *g* be continuous and let the pair (*f*, *g*) be weakly increasing. Then, *f* and *g* have a common fixed point z* in *X*.

**Proof**

Let *x*_0_ ∈ *X*. Let {*x*_*n*_} in *X* be constructed such that *x*_2*n*+1_ = *fx*_2*n*_ and *x*_2*n*+2_ = *gx*_2*n*+1_, for all nonnegative integers *n*. As *f* and *g* are weakly increasing, we have

(100) x1=fx0⪯gfx0=x2=gx1⪯fgx1=x3⪯⋯⪯x2n+1=fx2n⪯gfx2n=x2n+2⪯….

Following the proof of the above theorem there exists z* ∈ *X* such that

(101) $$\begin{array}{c} \underset{n\to \infty }{\lim}\sigma_{b}\left(x_{2n+1},z^{*}\right)=\underset{n\to \infty }{\lim}\sigma_{b}\left(fx_{2n},z^{*}\right)=0,\\ \underset{n\to \infty }{\lim}\sigma_{b}\left(x_{2n+2},z^{*}\right)=\underset{n\to \infty }{\lim}\sigma_{b}\left(gx_{2n+1},z^{*}\right)=0. \end{array}$$

Using the triangular inequality, we get

(102) σb(z∗,fz∗)≤sσb(z∗,fx2n)+sσb(fx2n,fz∗),σb(z∗,gz∗)≤sσb(z∗,gx2n+1)+sσb(gx2n+1,gz∗).

Letting *n* → *∞* and using continuity of *f* and *g*, we get

(103) σb(z∗,fz∗)≤slim⁡n→∞σb(z∗,fx2n)+slim⁡n→∞σb(fx2n,fz∗)=sσb(fz∗,fz∗)≤2sσb(fz∗,gz∗),σb(z∗,gz∗)≤slim⁡n→∞σb(z∗,gx2n+1)+slim⁡n→∞σb(gx2n+1,gz∗)=sσb(gz∗,gz∗)≤2sσb(fz∗,gz∗).

Therefore,

(104) max⁡{σb(z∗,fz∗),σb(z∗,gz∗)} ≤max⁡⁡{sσb(fz∗,fz∗),sσb(gz∗,gz∗)} ≤2sσb(gz∗,fz∗).  

From (98), we have

(105) $$\begin{array}{c} \psi \left(2s\sigma_{b}\left(fz^{*},gz^{*}\right)\right)\leq \psi \left(M\left(z^{*},z^{*}\right)\right)-\varphi \left(M\left(z^{*},z^{*}\right)\right), \end{array}$$

where

(106) M(z∗,z∗)=max⁡{σb(z∗,z∗),σb(z∗,fz∗),σb(z∗,gz∗),σb(z∗,gz∗)+σb(z∗,fz∗)4s}=max⁡{σb(z∗,fz∗),σb(z∗,gz∗)}.

Hence, by (104) and (105), we get that *φ*(*M*(z*, z*)) = 0. Thus, we have fz* = gz* = z*.

### 3.3. Common Fixed Points of Dominated Maps on Closed Balls

Motivated by [32, Theorem 2.1] we have the following result.

**Theorem 27**

Let (*X*, ⪯, *σ*_*b*_) be a complete ordered *b*-metric-like space, *S*, *T* : *X* → *X* dominated maps, and *x*_0_ an arbitrary point in *X*. Suppose that for *k* ∈ [0, 1/*s*) and for *S* ≠ *T* one has

(107) σb(Sx,Ty)≤kσb(x,y)for  all  comparable  elements  x,y  in  B(x0,rs)¯,

(108) $$\begin{array}{c} \sigma_{b}\left(x_{0},Sx_{0}\right)\leq \left(1-ks\right)r. \end{array}$$

Let, for each nonincreasing sequence {*x*_*n*_} in $\bar{B(x_{0},rs)}$, {*x*_*n*_} → *u* imply that *u*⪯*x*_*n*_. Then there exists $x^{*}\in \bar{B(x_{0},rs)}$ such that *σ*_*b*_(x*, x*) = 0 and x* = Sx* = Tx*. Also, if for any two points *x*, *y* in $\bar{B(x_{0},rs)}$ there exists a point $z\in \bar{B(x_{0},rs)}$ such that *z*⪯*x* and *z*⪯*y*, that is, every pair of elements has a lower bound, then x* is a unique common fixed point in $\bar{B(x_{0},rs)}$.

**Proof**

Choose a point *x*_1_ in *X* such that *x*_1_ = *Sx*_0_. As *Sx*_0_⪯*x*_0_, so *x*_1_⪯*x*_0_ and let *x*_2_ = *Tx*_1_. Now *Tx*_1_⪯*x*_1_ gives *x*_2_⪯*x*_1_, and continuing this process, we construct a sequence *x*_*n*_ of points in *X* such that

(109) x2i+1=Sx2i,  x2i+2=Tx2i+1,  x2i+1=Sx2i⪯x2iwhere  i=0,1,2,….

First we show that $x_{n}\in \bar{B(x_{0},rs)}$ for all *n* ∈ *N*. Using inequality (108), we have

(110) $$\begin{array}{c} \sigma_{b}\left(x_{0},x_{1}\right)\leq \left(1-ks\right)r\leq rs. \end{array}$$

It follows that $x_{1}\in \bar{B(x_{0},rs)}$.

Let $x_{2},\ldots ,x_{j}\in \bar{B(x_{0},rs)}$ for some *j* ∈ *N*. If *j* = 2*i* + 1, then *x*_2*i*+1_⪯*x*_2*i*_, where *i* = 0,1, 2,…, (*j* − 1)/2 so, using inequality (107), we obtain

(111) σb(x2i+1,x2i+2) =σb(Sx2i,Tx2i+1)≤k[σb(x2i,x2i+1)] ≤k2[σb(x2i−1,x2i)]≤⋯≤k2i+1σb(x0,x1).

If *j* = 2*i* + 2, then as $x_{1},x_{2},\ldots ,x_{j}\in \bar{B(x_{0},rs)}$ and *x*_2*i*+2_⪯*x*_2*i*+1_ (*i* = 0,1, 2,…, (*j* − 2)/2) we obtain

(112) $$\begin{array}{c} \sigma_{b}\left(x_{2i+2},x_{2i+3}\right)\leq k^{2(i+1)}\sigma_{b}\left(x_{0},x_{1}\right). \end{array}$$

Thus from inequalities (111) and (112), we have

(113) $$\begin{array}{c} \sigma_{b}\left(x_{j},x_{j+1}\right)\leq k^{j}\sigma_{b}\left(x_{0},x_{1}\right). \end{array}$$

Now

(114) σb(x0,xj+1) ≤sσb(x0,x1)+s2σb(x1,x2)+⋯+sjσb(xj−1,xj)  +sjσb(xj,xj+1) ≤sσb(x0,x1)+ks2σb(x0,x1)+⋯+kj−1sjσb(x0,x1)  +kjsjσb(x0,x1) (by(113)) ≤sσb(x0,x1)[1+ks+⋯+kj−1sj−1+kjsj] ≤s(1−kj+1sj+1)1−ksσb(x0,x1) ≤s(1−kj+1sj+1)1−ks(1−ks)r (by (108)) ≤s(1−kj+1sj+1)r≤rs.

Thus, $x_{j+1}\in \bar{B(x_{0},rs)}$. Hence $x_{n}\in \bar{B(x_{0},rs)}$ for all *n* ∈ *N*. This implies that

(115) $$\begin{array}{c} \sigma_{b}\left(x_{n},x_{n+1}\right)\leq k^{n}\sigma_{b}\left(x_{0},x_{1}\right),\;\forall n\in N. \end{array}$$

It follows that

(116) σb(xn,xn+i) ≤sσb(xn,xn+1)+s2σb(xn+1,xn+2)  +⋯+si−1σb(xn+i−2,xn+i−1)+si−1σb(xn+i−1,xn+i) ≤sknσb(x0,x1)+s2kn+1σb(x0,x1)  +⋯+si−1ki−2σb(x0,x1)+si−1kn+i−1σb(x0,x1) (by (115)) ≤sknσb(x0,x1)[1+ks+⋯+ki−2si−2+ki−1si−2] ≤skn(1−kisi)1−ksσb(x0,x1)⟶ as  n⟶∞.

Notice that the sequence {*x*_*n*_} is a Cauchy sequence in $\left(\bar{B(x_{0},rs)},\sigma_{b}\right)$. Therefore there exists a point $x^{*}\in \bar{B(x_{0},rs)}$ with lim⁡_*n*→*∞*_⁡*x*_*n*_ = x*. Also,

(117) $$\begin{array}{c} \underset{n\to \infty }{\lim}\sigma_{b}\left(x_{n},x^{*}\right)=0. \end{array}$$

Now,

(118) $$\begin{array}{c} \sigma_{b}\left(x^{*},Sx^{*}\right)\leq s\sigma_{b}\left(x^{*},x_{2n+2}\right)+s\sigma_{b}\left(x_{2n+2},Sx^{*}\right). \end{array}$$

On taking the limit as *n* → *∞* and using the fact that x*⪯*x*_*n*_ when *x*_*n*_ → x*, we have

(119) $$\begin{array}{c} \sigma_{b}\left(x^{*},Sx^{*}\right)\leq s\underset{n\to \infty }{\lim}\left[\sigma_{b}\left(x^{*},x_{2n+2}\right)+k\sigma_{b}\left(x_{2n+1},x^{*}\right)\right]. \end{array}$$

By (117) we obtain

(120) $$\begin{array}{c} \sigma_{b}\left(x^{*},Sx^{*}\right)\leq 0, \end{array}$$

and hence x* = Sx*. Similarly, by using

(121) $$\begin{array}{c} \sigma_{b}\left(x^{*},Tx^{*}\right)\leq s\sigma_{b}\left(x^{*},x_{2n+1}\right)+s\sigma_{b}\left(x_{2n+1},Tx^{*}\right), \end{array}$$

we can show that x* = Tx*. Hence *S* and *T* have a common fixed point in $\bar{B(x_{0},rs)}$. Now,

(122) $$\begin{array}{c} \sigma_{b}\left(x^{*},x^{*}\right)=\sigma_{b}\left(Sx^{*},Tx^{*}\right)\leq k\sigma_{b}\left(x^{*},x^{*}\right). \end{array}$$

This implies that

(123) $$\begin{array}{c} \sigma_{b}\left(x^{*},x^{*}\right)=0. \end{array}$$

For the uniqueness, assume that *y* is another fixed point of *T* and *S* in $\bar{B(x_{0},rs)}$. If x* and *y* are comparable then

(124) $$\begin{array}{c} \sigma_{b}\left(x^{*},y\right)=\sigma_{b}\left(Sx^{*},Ty\right)\leq k\sigma_{b}\left(x^{*},y\right). \end{array}$$

This shows that x* = *y*. Now if x* and *y* are not comparable then there exists a point $z_{0}\in \bar{B(x_{0},rs)}$ such that *z*_0_⪯x* and *z*_0_⪯*y*. Choose a point *z*_1_ in *X* such that *z*_1_ = *Tz*_0_. As *Tz*_0_⪯*z*_0_, so *z*_1_⪯*z*_0_; let *z*_2_ = *Sz*_1_. Now *Sz*_1_⪯*z*_1_ gives *z*_2_⪯*z*_1_, and continuing this process choose *z*_*n*_ in *X* such that

(125) z2i+1=Tz2i,  z2i+2=Sz2i+1,  z2i+1=Tz2i⪯z2iwhere  i=0,1,2,….

It follows that *z*_*n*+1_⪯*z*_*n*_⪯⋯⪯*z*_0_⪯x*. As *z*_0_⪯x* and *z*_0_⪯*y*, it follows that *z*_*n*_⪯Tx* and *z*_*n*_⪯*Ty* for all *n* ∈ *N*. We will prove that $z_{n}\in \bar{B(x_{0},rs)}$ for all *n* ∈ *N* by using mathematical induction. For *n* = 1,

(126) σb(x0,z1)≤sσb(x0,x1)+sσb(x1,z1)≤s(1−ks)r+ksσb(x0,z0)≤s(1−ks)r+ks2r=sr.

It follows that *z*_1_∈$\bar{B(x_{0},rs)}$. Let $z_{2},z_{3}\ldots ,z_{j}\in \bar{B(x_{0},rs)}$ for some *j* ∈ *N*. Note that if *j* is odd then

(127) σb(xj+1,zj+1)=σb(Txj,Szj)≤kσb(xj,zj)≤⋯≤kj+1σb(x0,z0),

and if *j* is even then

(128) σb(xj+1,zj+1)=σb(Sxj,Tzj)≤kσb(xj,zj)≤⋯≤kj+1σb(x0,z0).

Now,

(129) σb(x0,zj+1) ≤sσb(x0,x1)+s2σb(x1,x2)+⋯+sjσb(xj,xj+1)  +sjσb(xj+1,zj+1) ≤sσb(x0,x1)+ks2σb(x0,x1)+⋯+kjsjσb(x0,x1)  +kj+1sjσb(x0,z0) ≤sσb(x0,x1)[1+ks+⋯+kjsj−1]+kj+1sj+1r ≤sσb(x0,x1)[1+ks+⋯+kjsj]+kj+1sj+1r ≤s(1−ks)r(1−kj+1sj+1)1−ks+kj+1sj+1r =sr−kj+1sj+2r+kj+1sj+1r ≤sr−kj+1sj+2r+kj+1sj+2r=sr,

and this implies that

(130) $$\begin{array}{c} \sigma_{b}\left(x_{0},z_{j+1}\right)\leq sr. \end{array}$$

Thus $z_{j+1}\in \bar{B(x_{0},rs)}$. Hence $z_{n}\in \bar{B(x_{0},rs)}$ for all *n* ∈ *N*. As *z*_0_⪯x* and *z*_0_⪯*y*, it follows that *z*_*n*_⪯*T* ^*n*^ x*, *z*_*n*_⪯*S* ^*n*^ x*, *z*_*n*_⪯*S* ^*n*^ *y*, and *z*_*n*_⪯*T* ^*n*^ *y* for all *n* ∈ *N* as *S* ^*n*^ x* = *T* ^*n*^ x* = x* and *S* ^*n*^ *y* = *T* ^*n*^ *y* = *y* for all *n* ∈ *N*. If *n* is odd then

(131) σb(x∗,y) =σb(Tnx∗,Tny) ≤sσb(Tnx∗,Szn)+sσb(Szn,Tny) ≤ksσb(Tn−1x∗,zn)+ksσb(zn,Tn−1y) =ksσb(Sn−1x∗,Tzn−1)+ksσb(Tzn−1,Sn−1y) ≤k2sσb(Sn−2x∗,zn−1)+k2sσb(zn−1,Sn−2y) ⋮ ≤kn+1sσb(x∗,z0)+kn+1sσb(z0,y)⟶0 as  n⟶∞.

Hence, x* = *y*. Similarly, we can show that x* = *y* if *n* is even.

Thus, x* is a unique common fixed point of *T* and *S* in $\bar{B(x_{0},rs)}$.

### 3.4. Periodic Point Results

Clearly, a fixed point of *f* is also a fixed point of *f* ^*n*^ for every *n* ∈ *ℕ*; that is, *F*(*f*) ⊂ *F*(*f* ^*n*^). However, the converse is false. For example, the mapping *f* : ℝ → ℝ, defined by *fx* = 1/2 − *x*, has a unique fixed point 1/4 but every *x* ∈ ℝ is a fixed point of *f* ^2^. If *F*(*f*) = *F*(*f* ^*n*^) for every *n* ∈ *ℕ*, then *f* is said to have property *P*. For more details, we refer to [33, 34] and the references mentioned therein.

Recently, the study of periodic points for contraction mappings has been considered by many authors; for instance, every quasicontraction *f* : *X* → *X* with the constant *α* ∈ [0, 1/2), where *X* is a cone metric space, has the property *P* [35, Theorem 3.1] and if (*X*, *d*) is a cone metric space and *T*-Hardy-Rogers contraction *f* : *X* → *X* satisfies some appropriate conditions, then *f* has property *P* [36, Corollary 3.3].

**Definition 28 (see [21])**

Let (*X*, ⪯) be a partially ordered set. A mapping *f* is called dominating on *X* if *x*⪯*fx* for each *x* in *X*.

**Example 29 (see [21])**

Let *X* = [0, *∞*) be endowed with the usual ordering. Let *f* : *X* → *X* be defined by $fx=\sqrt[{n}]{x}$ for *x* ∈ [0,1) and *fx* = *x* ^*n*^ for *x* ∈ [1, *∞*), for any *n* ∈ *ℕ*. Then, for all *x* ∈ *X*, *x* ≤ *fx*; that is, *f* is a dominating map.

We have the following results.

**Theorem 30**

Let (*X*, ⪯, *σ*_*b*_) be a partially ordered complete *b*-metric-like space. Let *f* : *X* → *X* be a nondecreasing mapping such that for all *x* ∈ *X* with *x*⪯*fx* one has

(132) $$\begin{array}{c} \sigma_{b}\left(fx,f^{2}x\right)\leq \lambda \sigma_{b}\left(x,fx\right), \end{array}$$

where *λ* ∈ [0,1). Then *f* has the property *P* provided that *F*(*f*) is nonempty and *f* is dominating on *F*(*f* ^*n*^).

**Proof**

Let *u* ∈ *F*(*f* ^*n*^) for some *n* > 1. We will show that *u* = *fu*. Since *f* is dominating on *F*(*f* ^*n*^), therefore *u*⪯*fu* which implies that *f* ^*n*−1^ *u*⪯*f* ^*n*^ *u* as *f* is nondecreasing. Using (132), we obtain that

(133) σb(u,fu)=σb(ffn−1u,f2fn−1u)≤λσb(fn−1u,fnu)=λσb(ffn−2u,f2fn−2u).

Repeating the above process, we get

(134) $$\begin{array}{c} \sigma_{b}\left(u,fu\right)\leq \lambda^{n}\sigma_{b}\left(u,fu\right), \end{array}$$

which, on taking the limit as *n* → *∞*, implies that *σ*_*b*_(*u*, *fu*) = 0, implying that *u* = *fu*.

**Theorem 31**

Let *X* and *f* be as in Theorem 18. If *f* is dominating on *X*, then *f* satisfies property *P*.

**Proof**

From Theorem 18, *F*(*f*) ≠ *∅*. We will prove that (132) is satisfied for all *x*⪯*fx*.

If *σ*_*b*_(*fx*, *f* ^2^ *x*) = 0, then it is easy to see that (132) is satisfied. Now we suppose that *σ*_*b*_(*fx*, *f* ^2^ *x*) > 0. Since *f* is dominating, we have *x*⪯*fx*. Also, *fx*⪯*f* ^2^ *x* as *f* is nondecreasing. Using (15), we have

(135) σb(fx,f2x) =σb(fx,ffx) ≤αmax⁡{σb(x,fx),σb(x,fx),σb(fx,f2x), σb(x,f2x),σb(fx,fx)} =αmax⁡{σb(x,fx),σb(fx,f2x),σb(x,f2x), σb(fx,fx)} ≤αmax⁡{σb(x,fx),σb(fx,f2x),sσb(x,fx) +sσb(fx,f2x),2sσb(x,fx)}.

We will show that *σ*_*b*_(*fx*, *f* ^2^ *x*) ≤ *σ*_*b*_(*x*, *fx*). On the contrary, if *σ*_*b*_(*x*, *fx*) < *σ*_*b*_(*fx*, *f* ^2^ *x*), then from the above inequality we have

(136) $$\begin{array}{c} \sigma_{b}\left(fx,f^{2}x\right)<2s\alpha \sigma_{b}\left(fx,f^{2}x\right), \end{array}$$

which implies that 1 < 2*sα* < 2*s* · (1/2*s* ^2^) < 1, a contradiction.

So we have

(137) $$\begin{array}{c} \sigma_{b}\left(fx,f^{2}x\right)\leq \lambda \sigma_{b}\left(x,fx\right), \end{array}$$

where *λ* = 2*sα*. Obviously, *λ* ∈ [0,1). By Theorem 30, *f* has property *P*.
